# A Supervised Learning Process to Validate Online Disease Reports for Use in Predictive Models

**DOI:** 10.1089/big.2015.0019

**Published:** 2015-12-01

**Authors:** Helena M.M. Patching, Laurence M. Hudson, Warrick Cooke, Andres J. Garcia, Simon I. Hay, Mark Roberts, Catherine L. Moyes

**Affiliations:** ^1^ Tessella, Abingdon, United Kingdom.; ^2^ Bill & Melinda Gates Foundation, Seattle, Washington.; ^3^ Institute of Health Metrics and Analysis, University of Washington, Seattle, Washington.; ^4^ Spatial Ecology & Epidemiology Group, Wellcome Trust Centre for Human Genetics, University of Oxford, Oxford, United Kingdom.; ^5^ Tessella, Stevenage, United Kingdom.

**Keywords:** big data analytics, data acquisition and cleaning, machine learning, structured data

## Abstract

Pathogen distribution models that predict spatial variation in disease occurrence require data from a large number of geographic locations to generate disease risk maps. Traditionally, this process has used data from public health reporting systems; however, using online reports of new infections could speed up the process dramatically. Data from both public health systems and online sources must be validated before they can be used, but no mechanisms exist to validate data from online media reports. We have developed a supervised learning process to validate geolocated disease outbreak data in a timely manner. The process uses three input features, the data source and two metrics derived from the location of each disease occurrence. The location of disease occurrence provides information on the probability of disease occurrence at that location based on environmental and socioeconomic factors and the distance within or outside the current known disease extent. The process also uses validation scores, generated by disease experts who review a subset of the data, to build a training data set. The aim of the supervised learning process is to generate validation scores that can be used as weights going into the pathogen distribution model. After analyzing the three input features and testing the performance of alternative processes, we selected a cascade of ensembles comprising logistic regressors. Parameter values for the training data subset size, number of predictors, and number of layers in the cascade were tested before the process was deployed. The final configuration was tested using data for two contrasting diseases (dengue and cholera), and 66%–79% of data points were assigned a validation score. The remaining data points are scored by the experts, and the results inform the training data set for the next set of predictors, as well as going to the pathogen distribution model. The new supervised learning process has been implemented within our live site and is being used to validate the data that our system uses to produce updated predictive disease maps on a weekly basis.

## Introduction

Geographical maps of disease risk are used in many areas of public health. They can be combined with population surfaces to calculate the population at risk^1^; they are strong tools to advocate for resources where they are most needed,^2^ and they predict where new outbreaks are most likely.^3^ Surveillance data underpin these maps, but are typically incomplete; therefore, we use predictive pathogen distribution models to estimate risk at all locations. Producing these maps is a lengthy process and much of this time is taken up processing surveillance data before they go into the model.^4^

We have established a new project, the Atlas of Baseline Risk Assessment for Infectious Diseases (ABRAID), to reduce the time taken to generate predictive disease maps from 3 years to 3 weeks. This is achieved by using novel online data sources that report disease outbreaks (one or more new infections occurring at a specific location) within days of occurrence. Approaches to capturing this data have been exemplified by HealthMap^5^ that presents disease outbreaks as points on a global map through their website.

Traditional surveillance data require extensive checks and validation. This is even more important for rapidly produced data from online sources, but the process is potentially time-consuming. Our solution is to use machine learning techniques to validate data, specifically for use in pathogen distribution models, within a short time frame.

Our mapping system validates incoming data using a supervised learning process that targets those data with the largest potential impact on the disease risk map and supports our primary aim of tracking disease spread. Specifically, the process was designed to target disease reports located outside the current disease extent in areas predicted to be suitable for the disease, or located within the current extent, but in areas with a low predicted suitability. The first class of data may represent spread of the disease to new areas and the second may represent a change in the niche occupied; both will have a high impact on disease maps compared to occurrences in locations where the disease is already known to occur. Alternatively, these data may reflect inaccuracies in our current predictions or they may be invalid reports.

Each component of the system has been deployed in our live site, and this article describes the rationale, design, and testing of the machine learning process built to validate disease reports.

## Methods and Implementation

### Data coming into the system

Data are primarily obtained from a web service provided by HealthMap.^5,6^ HealthMap scans news media (e.g., Google News), expert-curated accounts (e.g., ProMED Mail), and other official web alerts, using text processing algorithms to classify reports by location and disease. Our system then links the data provided by HealthMap to point locations (<5 km^2^) or polygons (>5 km^2^). Our system has the flexibility to receive data from other sources, but the primary provider is HealthMap.

### Input features available for the learning task

**FIG. 1.**
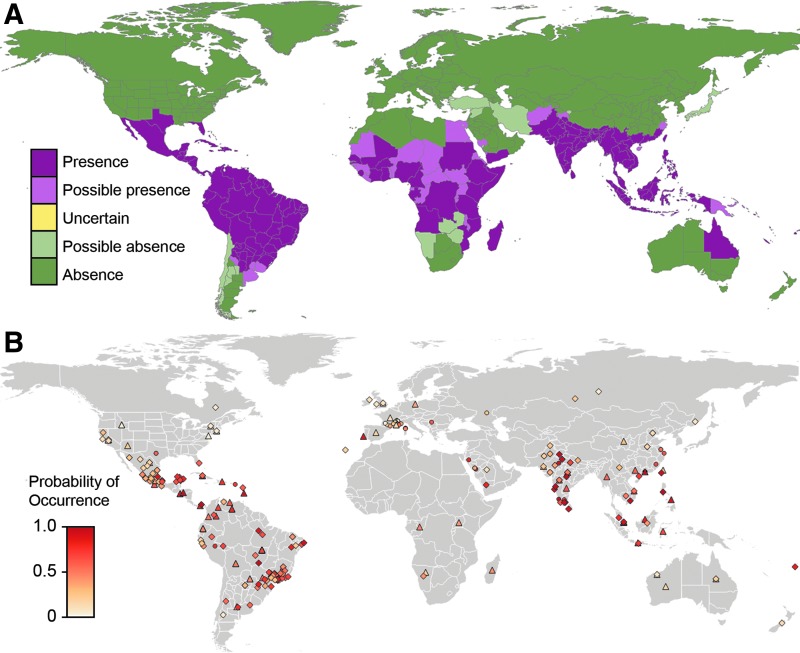
The input features linked to the dengue data set. The map in **(A)** shows the disease extent for dengue. The map in **(B)** shows the probability of occurrence for each data point; data extracted from ProMed reports are represented by a *diamond*, data extracted from Google News reports are represented by a *circle*, and data extracted from other feeds are represented by a *triangle*.

For each new disease occurrence, we know (1) the source feed of the report (e.g., Google News and ProMED Mail) and the location of the outbreak, which give us (2) the predicted probability of disease occurrence at that location as estimated by the pathogen distribution model, and (3) the distance from the current disease extent (positive values outside the extent boundary and negative values within). The three properties selected (probability of disease occurrence, distance from extent, and source feed) represent the input features to the learning task (Fig. 1).

Briefly, the pathogen distribution model uses environmental data (temperature, land cover types, etc.) and socioeconomic data (population density, accessibility, poverty, etc.), at disease report locations, plus data on absence and reporting bias, to define the relationship between the suite of potential covariates and the probability of disease occurrence.^7^ The model then extrapolates to areas without disease data and estimates the probability of one or more new infections occurring at each location.

### Generating a labeled training data set

For each new data point, we need to generate a metric representing its validity (used as a weighting in the pathogen distribution model). The inferred learning process generates this value using a training data set that has been labeled by disease experts. We asked eight experts to review each data point in the initial training data sets and respond “Invalid,” “Uncertain,” or “Valid” corresponding to the values 0, 0.5, or 1.0, respectively. We took the average of the values generated across the experts to obtain a measure between 0 and 1, which acts as the label or dependent variable in the supervised learning task and is termed the “expert-derived validation score.” In the live system, external experts are given the same options plus the choice of “Don't know,” which is not linked to a value.

We used data for two contrasting diseases to design and test the process. Dengue fever is caused by a virus transmitted between humans by mosquitoes within the tropics. Cholera is a food-, fecal-, and water-borne bacterial disease with a global distribution. The two training data sets comprised 400 occurrences of dengue fever and 1036 occurrences of cholera, which were reviewed by disease experts, giving each data point an “expert-derived validation score.” The dengue data set was used for the initial analysis of the data distribution and to investigate and refine the optimal parameters of the devised machine learning process. Both data sets were used to test the final configuration.

### Designing a machine learning process based on the training data set

**FIG. 2.**
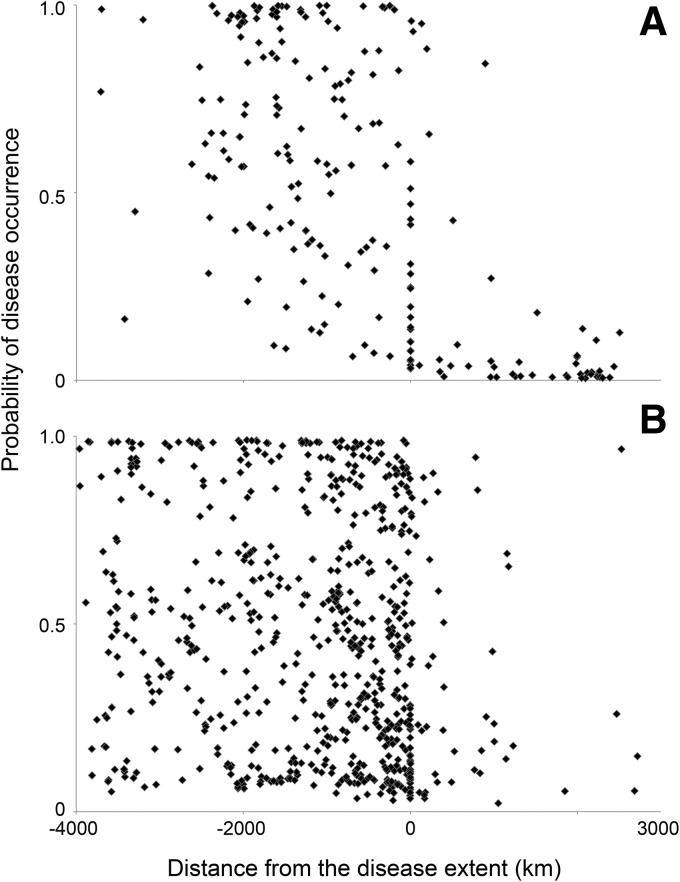
The distribution of the disease data when distance from disease extent was plotted against the probability of occurrence. Positive “distance from disease extent” values fall outside the extent boundary (in areas where the disease is currently considered absent) and negative values within. The plot in **(A)** shows the distribution of values for dengue. The plot in **(B)** shows the same distribution for cholera.

The spread of the training data set for dengue (Fig. 2) did not display an immediately obvious trend. We needed a method that could discover trends within subspaces of the full data range (e.g., the points within the extent may exhibit a different pattern to those outside the extent), yet be flexible to detecting these subspaces from the data, rather than having to manually discern or define them per disease. Therefore, we devised a multistage “cascade,”^8–10^ where the first predictor model (*P_i_*) is trained on all available data, then the uncertain points in that set (for which a trusted prediction could not be made by that model) become the training set for the model in the next “layer*”* of the cascade (*P_i_*_+1_), and so on, until some stopping criteria are met (such as no data remain or a maximum number of layers *L* is reached). These models are configured identically using the same features in the data sets and the same number of logistic regression models. The only difference being the subset of data they are trained on.

To determine which points fall through to the next layer, we must quantify uncertainty in the prediction from the layer as well as the predicted value itself. This is achieved using an ensemble of predictors. A layer comprises *m* predictors, $P_{ij} \ { \rm for} \ j = 1.m$. Each predictor, *P_ij_*, is trained on a different randomly selected subset (*p*%) of the training data for that specific layer.^11^ The overall prediction for the layer ($y_i { \prime}$) is taken as the mean of the *m* values, if the extrinsic uncertainty, namely the coefficient of variation (CV) of the *m* values, is below some threshold, *c*.

During the prediction step, every new data point continues through the layers until a reliable prediction for that point is made, otherwise it will be reviewed by the experts and then added to the training data set.

### Parameterizing the machine learning process

We randomly split the available data for dengue into 90% training data and 10% testing data.^12^ This division was selected to reflect the target split for the deployed system, that is, the proportion of points we could reasonably ask experts to manually validate. We varied the parameters of the ensemble cascade structure and investigated the effect to select the optimum values that would maximize accuracy of prediction while ensuring a manageable proportion of data points go for expert review.

To select the predictor type, we used an initial set of parameters (*m* = 5 predictors in each layer, trained on *p* = 50% of the data in the layer, with a CV threshold of 0.05, and stopping condition that the number of layers *L* does not exceed 40). Three variants of the ensemble cascade structure were constructed with 90% of the available data set: one where all units in the layers are Support Vector Machines (SVM)^13^ (using the radial basis function [RBF] kernel and regularization parameter C = 1e2); one with k-nearest neighbor (k-NN with *k* = 3 and a uniform weights function) regressors; and one with logistic regression models.^14^ In each case, we compared the mean absolute error between overall predicted value, $y{ \prime}$, and actual expert-derived score, *y*, for the 10% test set, as well as identifying the number of occurrences in the test data set resulting in no prediction.

Other kernel functions and parameters were tested, but did not improve the results for error or goodness of fit. Briefly, we varied the regularization parameter C and reviewed the effect on the error and the goodness of fit of the model. Using a linear kernel and using a two-degree polynomial kernel resulted in similar performance as the RBF kernel, and a sigmoidal kernel function performed the worst. There was no improvement for varying C, and the test root mean square error (RMSE) was never <0.43.

To test how the subset size (*p%* used by each unit in each layer) affects the predictions, we trained one ensemble layer with varying proportions of the 90% training set. Then, for all the points in the 10% test set, we calculated the mean CV $\left( \frac { \sigma_1 }  { \mu_1 } \right)$ of the *m* = 5 predicted values and the mean error between the true label from experts and each of the 5 values $( \frac { 1 }  { m } \sum \nolimits^m_ { j = 1 } abs ( y - y_ { 1j } ) )$. Since the proportion is a random subset each time, we repeated this process 10 times and viewed the distribution and the averages of these metrics over the 10 iterations.

Similarly, now holding *p* at 40%, we varied the number of predictors in one layer, *m* from 1 to 20, and examined how the CV of the *m* values and the mean error to the predictions changed.

### Testing the machine learning process

The data sets for dengue and cholera were split and used to train the final configuration of the ensemble cascade 128 times each, using the parameters determined during the steps mentioned earlier, to test its performance, giving an unbiased estimate of generalization error.

Testing the system using the dengue data, we used a training set of 200 occurrences (set $A = \{  ( x , y ) \}$) to train the predictor for this disease and a test set of 200 data points (set $B = \{  ( x , y , y{ \prime} ) \}$). All occurrences were validated by experts and assigned a true validation score, *y*. We can compare this value against the prediction $y{ \prime}$ obtained for each occurrence in B. This was repeated using cholera data with 641 occurrences (set C) used to train the predictor and a test set of 365 data points (set D).

## Results

### Selecting a predictor type

The ensemble cascade generated for logistic regression models showed the greatest accuracy (test error was 0.08, compared to 0.13 for SVM and 0.10 for k-NN), with an acceptable proportion of occurrences falling through to the final layer (approximately one quarter) and an appropriate resultant number of layers (12 on average). When all the units in the ensembles were k-NN regression models, approximately ¾ of the occurrences were assigned a validation score from only the first layer. Using SVM models resulted in an overly complex structure with arguably too many layers (20–40), since only a small subset of predictions could be accurately made on each layer, and enforcing a limit on the number of layers meant that over half the points were not assigned scores. We therefore selected a logistic regression model.

The predicted score needs to have a bounded output between 0 and 1, as prescribed by the pathogen distribution model, meaning that a linear regression would be unsuitable. The training labels are always within this range, but with this method there is no constraint on the value output by SVM or k-NN. Predictions with these methods were observed to be outside the range and often orders of magnitude larger. The sigmoidal logistic loss function is more appropriate, since it displays the desired behavior and property of converging asymptotically to 0 and 1,^15^ and is less prone to overfitting.^14^

### Selecting the machine learning process parameters

As the subset size approaches 100% then CV tends to 0 and the average error decreases; each predictor receives progressively more information from the data set and more of the same information as the other predictors, until all predictors receive the same data and therefore return almost the same response. We conclude that a subset size in the region of 30%–40% satisfies the compromise between gaining useful variation in responses for this important metric of uncertainty and keeping accuracy (error between 0.12 and 0.13). We were reassured that even with a subset size of only 10%, the average error was not larger than 0.18.

**FIG. 3.**
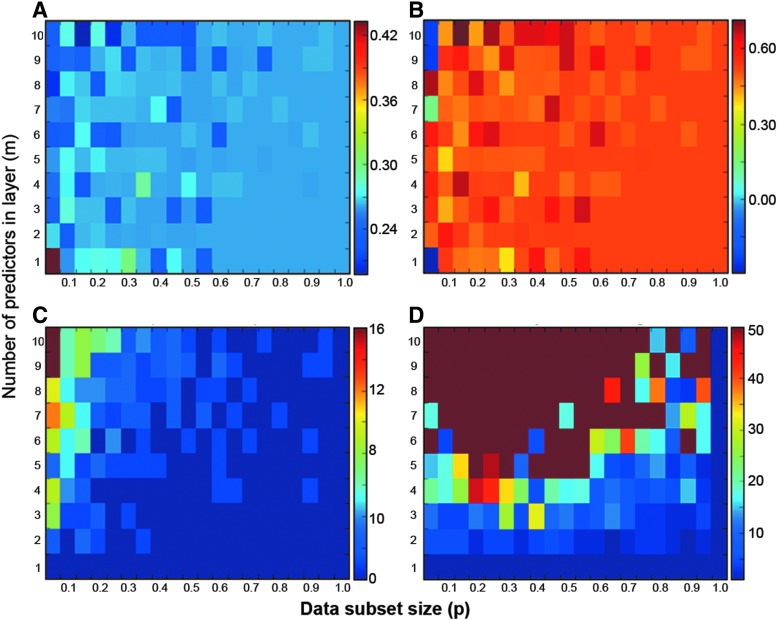
The four heatmaps show **(A)** the root mean square error (RMSE), **(B)** the coefficient of determination (*R*^2^), **(C)** the number of points without prediction, and **(D)** the number of layers in the resulting casade, when the number of predictors in each layer (*m*) and the data subset size (*p*) are varied. Our requirements for the combination of *m* and *p* were a low test RMSE, high *R*^2^, with reasonable number of points without prediction that go to the experts, and fewer layers.

After initial adjustment while *m* < 5, as *m* increases the average error settles around 0.1 and average CV plateaus in the region 0.03–0.06. We found that increasing the number of predictors in a layer, *m*, causes the CV of predictions to increase enough that the CV threshold, *c*, must also be increased, otherwise the number of layers in the resulting cascade structure increases dramatically. Therefore, the number of predictors in the layer should be set at a low value (5 or 6) to avoid increasing complexity without losing accuracy. Heatmaps generated by a grid search, in which both parameters are varied independently, are shown in Figure 3.

**FIG. 4.**
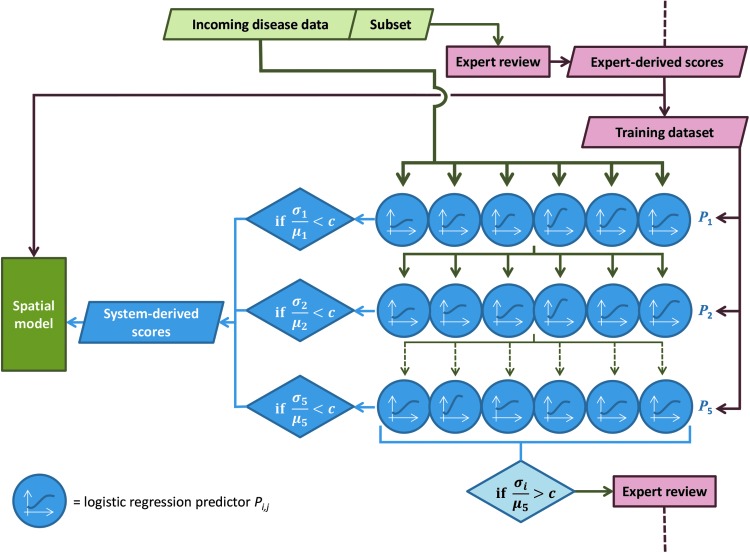
Diagram showing the process deployed.

To summarize, the most suitable configuration of the ensemble cascade was assessed to be *m* = 6 logistic regression models in each layer, each trained on a random *p* = 40% of the data in that layer, with a maximum of *L* = 5 layers (Fig. 4). The threshold on CV between the six predictions, to determine whether the values are sufficiently close to be accepted, was chosen as *c* = 0.05.

### Machine learning process performance

**FIG. 5.**
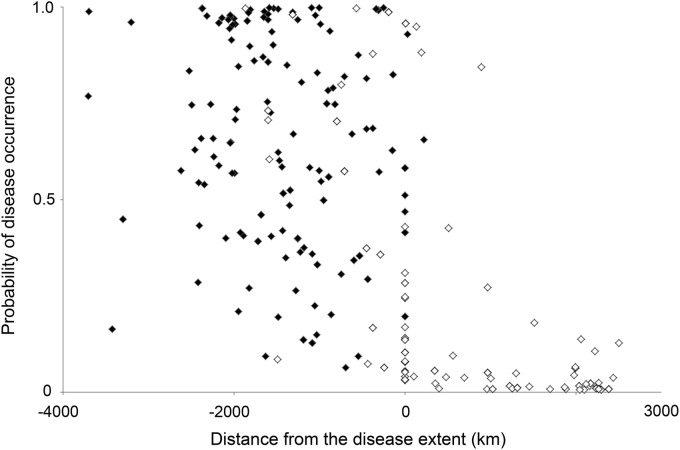
The distributions of data points assigned a score by the machine learning process (*closed diamonds*) and data points that the process did not assign a score (*open diamonds*).

On average, over the 128 iterations of ensemble cascade construction during the testing phase, 66% of occurrences in the dengue test set (Set B) were assigned a predicted score. The majority of data points that did not receive a predicted score, and would therefore be referred to the experts, were those located outside the disease extent (Fig. 5). The average test RMSE was 0.242. For cholera, on average 79% of occurrences in the test set (Set D) were assigned a predicted score during the testing phase, and the average test RMSE was 0.285.

The RMSE statistic reports how closely the fitted model is to the data points, in the same units or scale as the prediction, while amplifying and penalizing large errors.^16^ For context, a reasonably accurate predictor, whose random error is distributed uniformly between −0.1 and 0, results in an observed average RMSE of 0.057 over 100 iterations, and for a naive “bad” predictor where the prediction is any randomly generated value between 0 and 1 (again uniformly distributed), the average RMSE was 0.501. If the prediction is always 0.5, average RMSE was 0.411.

### Deployment of the machine learning process

The results described earlier determined the supervised learning process that was deployed. The interface that allows external experts to validate new data points was also deployed (www.abraid.ox.ac.uk/datavalidation) to ensure that the training data set is kept up-to-date. A subset of incoming data points is always sent to the experts, in addition to the data points that were not assigned a reliable score by the predictors. Each time new data come into the system, the expert validation scores from the preceding 12 months are used to generate a new training data set.

## Discussion

We have implemented a process that is flexible to different diseases, automatically adjusts with the data over time, and is able to filter questionable occurrences for manual review appropriately. The process deployed will be reevaluated as new diseases are incorporated, including leishmaniasis, Crimean Congo hemorrhagic fever, chikungunya, and melioidosis. Potential avenues to improve performance include strategies to address geographic sampling bias^17^ in the outbreak data, either by resampling the data in a bid to equalize the proportion of two classes or by adjusting the learning algorithm to handle the disproportion.^18^

This work has combined well-studied methods from different disciplines into a novel and fully automated end-to-end disease modeling system. Gammerman^19^ describes the ensemble approach we have used as a “conformal predictor” in that our “hedged” predictions from each layer of the cascade “include a quantitative measure of their own accuracy and reliability.” A similar case of a screening system in medicine has been devised to declare a patient disease free, if confident, or refer the test results to a human doctor.^20^

The primary aim of our mapping system is to track the spread of diseases to new areas, and the current supervised learning approach supports this aim. It focuses on data points that have the highest potential impact on the risk map because these data fall outside the current extent and/or in areas currently predicted to be unsuitable. A secondary aim is to track the shrinking distribution of diseases that is being eliminated, such as polio that is disappearing from areas where it was previously found. In this context, the ability to distinguish invalid data points that fall within areas where the disease was previously known to occur becomes important. The current system does not have access to information that distinguishes these data points. The next stage of this work requires new input data derived from the content of the original online report and will use natural language processes to identify reports of disease absence or elimination rather than presence.

Many groups have used data captured from the Internet, including social media, to analyze temporal trends in diseases such as influenza, characterized by large spikes of incidence, to detect outbreaks more quickly than traditional surveillance.^21,22^ Rather than using Internet data to predict outbreaks, we are using these data to model the baseline geospatial variation in global disease risk and we believe we are the first group to do so.

## Conclusion

This novel use of a supervised learning process is now operational and is, to our knowledge, the first time that data from online news media have been processed using supervised learning techniques for use in an epidemiological model. This is an open source and open access project; the source code is available at https://github.com/SEEG-Oxford/ABRAID-MP and the validated disease outbreak data and resulting spatial risk data are available from our website (www.abraid.ox.ac.uk).

## Abbreviations Used

CV: coefficient of variation
k-NN: k-nearest neighbour
RBF: radial basis function
RMSE: root mean square error
SVM: support vector machines
